# Heavy Metal Contamination in Rice-Producing Soils of Hunan Province, China and Potential Health Risks

**DOI:** 10.3390/ijerph121215005

**Published:** 2015-12-08

**Authors:** Fanfu Zeng, Wei Wei, Mansha Li, Ruixue Huang, Fei Yang, Yanying Duan

**Affiliations:** Department of Occupational and Environmental Health, School of Public Health, Central South University, Changsha 410078, China; zengff66@163.com (F.Z.); ww900222@126.com (W.W.); 18711505358@163.com (M.L.); arsenite@163.com (R.H.); phfyang@csu.edu.cn (F.Y.)

**Keywords:** heavy metal, soil-rice system, risk assessment

## Abstract

We studied Cd, Cr, As, Ni, Mn, Pb, and Hg in three agricultural areas of Hunan province and determined the potential non-carcinogenic and carcinogenic risks for residents. Soil and brown rice samples from Shimen, Fenghuang, and Xiangtan counties were analyzed by atomic absorption spectroscopy. Soil levels of Cd and Hg were greatest, followed by As and Ni. The mean concentrations of heavy metals in brown rice were Cd 0.325, Cr 0.109, As 0.344, Ni 0.610, Mn 9.03, Pb 0.023, and Hg 0.071 mg/kg, respectively. Cd and Hg had greater transfer ability from soil to rice than the other elements. Daily intake of heavy metals through brown rice consumption were estimated to be Cd 2.30, Cr 0.775, As 2.45, Ni 4.32, Pb 0.162, Mn 64.6 and Hg 0.503 µg/(kg·day), respectively. Cd, Hg and As Hazard Quotient values were greater than 1 and Cd, Cr, As and Ni Cancer Risk values were all greater than 10^−4^. The total non-carcinogenic risk factor was 14.6 and the total carcinogenic risk factor was 0.0423. Long-term exposure to heavy metals through brown rice consumption poses both potential non-carcinogenic and carcinogenic health risks to the local residents.

## 1. Introduction 

Heavy metals, such as Cd, Cr, Ni, and Hg, from mining, industrial processing and wastewater release are common contaminants of arable soil [1,2]. Zhao [3] found that 99% of the paddy soil samples from Nanxun county had Cd levels exceeding the natural background value indicating widespread Cd accumulation in the local soils. Mohanty [1] reported mean Cr levels of 11,170 mg/kg in the surface soils (0–20 cm) of an Indian mine area. Marwa [4] showed that soil samples in Tanzania contain up to 136.8 mg/kg of Ni, which exceeds the maximum tolerance level set by the European Economic Community [5]. 

Certain heavy metals in agricultural soils are, at low levels, essential for plant growth [6], but some metals are highly toxic to humans. These toxic heavy metals can be absorbed and accumulated by plants and eventually enter the human body through food intake. Food consumption is the major exposure pathway, and exposure risk from ingestion exceeds risks from inhalation and dermal contact [7]. In Asian countries, rice (*Oryza sativa*) is a food staple used for daily consumption and provides over 70% of the energy derived from daily food intake [8]. Rice is the main source of heavy metal exposure, especially for inhabitants of China and Japan [9,10]. Consumption of rice contaminated with heavy metals is closely related to health impact. There is clear evidence that human renal dysfunction is linked to contamination of rice with Cd in China and Japan [11,12].

Hunan province in southern China is rich in mineral resources. The world’s largest arsenic disulfide mine is located in Shimen County, northwest of Hunan. The Huayuan-Fenghuang Pb/Zn mine, opened in 2012, is expected to be the largest Pb/Zn mine in China. There are many smelters and refineries in this province. Activities such as mining, waste, and slag disposal have contributed to soils polluted with heavy metals [13,14]. Hunan province is the main rice production area in southern China. The food safety issue of heavy metals has attracted substantial attention recently due to the discovery of “Cd rice” [15] as well as documentation of a high incidence of malignant tumors among the population living in heavy metal polluted areas [16,17].

Toxic metals such as Cd, As, and Pb occur in the rice and paddy soils of Hunan province [18,19,20]. These studies mainly focused on a single element or single sampling site. Carcinogenic heavy metals such as Cd, Cr, As, and Ni [21] and those with potential nervous system toxicity such as Mn, Hg, and Pb [22,23] have not been systematically analyzed and information on individual and combined health risk factors of these elements are scarce.

One objective of the present study was to conduct a risk assessment of seven toxic heavy metals (Cd, Cr, As, Ni, Pb, Mn and Hg). Concentrations of these heavy metals were determined in soil and brown rice from three areas to determine pollution levels and transfer factors. Risk assessment was made of the potential carcinogenic and non-carcinogenic risk factors for local residents consuming brown rice.

## 2. Materials and Methods

### 2.1. Study Area and Sampling

A total of 28 pairs of rice grain and soil samples were collected from Shimen (SM) (n = 10), Fenghuang (FH) (n = 10) and Xiangtan (XT) (n = 8) counties during the 2013 harvest season. Each soil sample (from 0 to 15 cm depth) was a composite of at least 5 sub-samples within a distance of 10 m surrounding a specific sampling location. Rice grain was grown in the corresponding soil sampling site and bought from local peasants. We collected at least 2 kg soil for each soil sample and 1 kg rice grain for each rice sample.

### 2.2. Sample Preparation and Analysis

Soil samples were air-dried in the laboratory for several days at ambient temperature. They were sieved through a 0.149 mm (100 mesh) nylon screen for digestion. Rice grain samples were oven-dried at 105 °C for 1 h, then at 70 °C to constant weight. Husks were removed. Then, the grain brown rice samples were comminuted to pass through a 100 mesh sieve and stored in closed polyethylene bags for digestion.

Total heavy metal concentrations in soil and brown rice were determined following digestion using strong acids of HF, HNO_3_ and HClO_4_. Cd and Pb concentrations were measured using graphite furnace atomic absorption spectroscopy (TAS-990 Super, Beijing, China) with 2% NH_4_H_2_PO_4_as its matrix modifier. Mn, Cr and Ni concentrations were determined by flame-atomic absorption spectroscopy (TAS-990 Super). As and Hg levels were assayed by hydride atomic absorption spectrometry (TAS-990 Super).

The accuracy of determinations was verified using the Chinese standardized reference materials (GSS-5, GSS-8 for soil samples; GBW (E) 080684 for rice samples). All samples were measured in duplicate. The recoveries of reference elements were within ±15% of the actual values.

### 2.3. Transfer Factor

Transfer factor (TF) is one of the main parameters of human exposure to toxic heavy metals through the food chain [24]. It is defined as the ratio of metal concentration(fresh weight) in the plant to the total metal concentration in the soil (dry weight) [25] was used to determine the transfer ability of heavy metals from soil to rice as well as to estimate their potential human health risks. The TF was computed as:

(1) $$TF=\frac{Cr}{Cs}$$

where Cr and Cs represent the heavy metal concentration in extracts of rice and soil, respectively.

### 2.4. Daily Intake Estimate of Heavy Metals through Brown Rice Consumption

The average daily intake (ADI, mg/(kg·day)) is used to quantify the oral exposure dosage for deleterious substances and was estimated using the following equation [18].

(2) $$ADI=\frac{C\times IR\times ED\times EF}{BW\times AT}$$

where C, IR, ED, EF, BW, and AT represent heavy metal content (mg/kg), ingestion rate, exposure duration, exposure frequency, reference body mass, and average time, respectively. The average daily rice intake of adults in Hunan province was estimated to be 0.425 kg [26]. The mean body weight was 58.1 kg based on measurements of 158,666 Chinese from all provinces [27]. Values of these parameters are listed in Table 1.

**Table 1:** Values of parameters in Equation 1.

Parameter	Value
IR	0.425 kg/d ^a^
ED	365 d
EF	74 a ^b^
BW	58.1 kg
AT	27,010 d ^c^

Notes: IR = ingestion rate; ED = exposure duration; EF = exposure frequency; BW = body weight; AT = average time; ^a^ d is the abbreviation for day; ^b^ a is the abbreviation for age; ^c^ 27,010 d = 365 d × 74 a.

### 2.5. Human Health Risk Assessment 

#### 2.5.1. Non-Carcinogenic Risk 

The human non-carcinogenic risk assessment for local people was calculated using reference dose (RfD) previously established by the Joint FAO (Food and Agriculture Organization)/WHO (Word Health Organization) Expert Committee on Food Additives (JECFA) [28] and the United States Environmental Protection Agency (USEPA) [29]. The hazard quotient (HQ), a ratio of average daily intake (ADI) and reference dose (RfD) [30], characterizes the health risk of non-carcinogenic adverse effects due to exposure to toxicants:

(3) HQ=ADI/RFD

where RfD is the estimated maximum permissible dose for human through daily exposure. If HQ < 1, adverse health effects would be unlikely experienced, whereas potential non-carcinogenic effects would occur when HQ ≥ 1 [31].The hazard index (HI) is calculated to evaluate the potential risk of adverse health effects from a mixture of chemical elements in brown rice. The HI was calculated as the sum of HQ [32] (assuming additive effects):

(4) HI=∑HQ

If HI < 1, chronic risks are assumed to unlikely happen, whereas non-cancer risks are likely to occur in case HI ≥ 1 [19]. 

#### 2.5.2. Carcinogenic Risk

The cancer risk (CR) was calculated by multiplying the average daily intake (in mg/(kg·day) over a lifetime) with a cancer slope factor (SF) according to Equation (4). CR is estimated as the incremental probability of an individual developing cancer over a lifetime. For example, a CR of 10^−4^ indicates a probability of 1 in 10,000 individuals developing cancer [33]. The CR of local people caused by potential carcinogen exposure over a lifetime was calculated according to the following equation [30]:

(5) CR=ADI×SF

(6) CRt=∑CR

If multiple carcinogenic elements are present, the cancer risks from all carcinogen are summed (assuming additive effects). Risks in the range of 1.0 × 10^−6^ to 1.0 × 10^−4^ are acceptable [19]. 

Cr, Cd, As and Ni were treated as potential carcinogenic contaminants, whereas Pb, Hg and Mn were regarded as non-carcinogenic elements, based on the order of classification group defined by the International Agency for Research on Cancer [21]. Table 2 shows the oral RfD and SF value is for heavy metals in food.

**Table 2:** Reference doses (RfD) and slope factors (SF) of seven heavy metals.

Elements	Classification by IARC ^a^	RfD (mg/kg·d)	Source	SF (mg/kg·d)^−1^	Source
Cd	1	1.00 × 10^−3^	IRIS ^b^	15	CALEPA^c^
Cr	1	1.5	IRIS	0.5	CALEPA
As	1	3.00 × 10^−4^	IRIS	1.5	IRIS
Ni	1	0.02	IRIS	0.91	CALEPA
Pb	2B	3.6 × 10^−3^ ^e^	WHO ^d^	-	CALEPA
Mn	-	0.14	IRIS	-	
Hg	3	1.60 × 10^−4^	CALEPA(WHO)	-	

Notes: ^a^ International Agency for Research on Cancer, group 1 chemicals are definite human carcinogens, group 2B chemicals are possible human carcinogens, and group 3 chemicals are non-carcinogenic; ^b^ Integrated Risk Information System, U.S. EPA; ^c^ California Environmental Protection Agency, U.S; ^d^ World Health Organization, WHO; ^e^ Calculated according to the provisional tolerable weekly intake, 25 µg/kg BW, WHO/FAO (1999).

## 3. Results

### 3.1. Heavy Metal Concentrations in Soil

Heavy metal concentrations in agricultural soils are shown in Table 3. The mean values of Cd and Hg were approximately five and 30 times higher than the corresponding maximum allowable concentration (MAC) [34]. Although the concentration of As, Ni, and Pb were below the MAC, they were higher than the corresponding natural background values, indicating accumulation of As, Ni and Pb in the soil. Soil samples had levels of Cr and Mn lower than their background values, suggesting no soil pollution with Cr and Mn in this area. 

**Table 3:** Concentrations (mean ± standard deviation, mg/kg) of seven elements in soil from three districts in Hunan province.

		Cd	Cr	As	Ni	Pb	Mn	Hg
SM	Mean ± S.D.	0.246 ± 0.098	26.0 ± 3.12	22.1 ± 14.9	36.2 ± 6.45	24.9 ± 3.63	317 ± 217	0.445 ± 0.168
(n = 10)	Range	0.062–0.436	23.2–33.5	3.16–45.2	26.6–48.7	19.8–32.3	123–752	0.199–0.684
FH	Mean ± S.D.	3.25 ± 8.71	23.7 ± 2.01	21.2 ± 6.40	36.5 ± 6.43	43.3 ± 21.1	278 ± 217	41.1 ± 58.5
(n = 10)	Range	0.039–28.01	21.5–28.2	12.2–34.8	25.9–44.5	19.6–83.6	96.7–833	1.58–56.8
XT	Mean ± S.D.	0.541 ± 0.386	33.1 ± 3.99	4.46 ± 1.19	27.7 ± 9.30	94.5 ± 76.7	476 ± 302	0.329 ± 0.227
(n = 8)	Range	0.18–1.17	28.1–39.3	3.30–6.31	14.9–40.0	37.7–262	189–865	0.123–0.689
Total	Mean ± S.D.	1.40 ± 5.22	27.2 ± 4.91	16.8 ± 12.3	33.9 ± 8.12	51.4 ± 50.1	348 ± 249	14.9 ± 39.2
	Range	0.039–28.0	21.5–39.3	3.16–45.2	14.9–48.7	19.6–262	96.7–865	0.123–192
Background value ^a^		0.098	68	14	31.9	27	459	0.096
	MAC ^b^	0.3	300	25	50	300	NV ^c^	0.5

Notes: ^a^ Soil Background Value in Hunan province and Study Methods (Pan *et al.* 1998); ^b^ MAC, maximum allowable concentration of heavy metals in soil, recommended by the Chinese Environmental Quality Standard for Soils, grade II(GB 15618-1995); ^c^ NV is the no criterion value.

Cd, As, and Hg soil levels showed significant variability among the three study areas. The maximum mean concentration of Cd was 3.25 mg/kg in FH. This was less than six times the levels found in SM and XT. Levels from SM and FH were less than five times greater than those from XT. The mean level of Hg in FH was 41.1 mg/kg and this exceeded values from SM and XT by ten times. These data indicate that the extent of pollution differs substantially among the three study areas.

### 3.2. Heavy Metal Concentrations in Brown Rice 

Heavy metal contents in brown rice samples are shown in Table 4. The values were consistent with the soil results. The means of Cd and Hg in brown rice were 0.312 and 0.069 mg/kg, respectively. Both values are greater than the national threshold value of food safety for Cd (0.2 mg/kg) and Hg (0.02 mg/kg) [35]. Mean Cd levels in brown rice from SM, FH, and XT were 0.281, 0.321, and 0.341 mg/kg and all were 1–2 times greater than the threshold value. For Hg, the mean brown rice concentrations from SM, FH, and XT were 0.043, 0.111, and 0.047 mg/kg, which were 2–5 times higher than the threshold value. 

**Table 4:** Concentrations (mean ± standard deviation, mg/kg) of seven elements in brown rice from three districts in Hunan province.

		Cd	Cr	As	Ni	Pb	Mn	Hg
SM	Mean ± S.D.	0.281 ± 0.275	0.081 ± 0.033	0.513 ± 0.348	0.677 ± 0.321	0.016 ± 0.005	8.38 ± 2.55	0.043 ± 0.013
(n = 10)	Range	0.006–0.716	0.029–0.113	0.272–1.148	0.283–1.221	0.009–0.025	4.94–14.19	0.025–0.059
FH	Mean ± S.D.	0.321 ± 0.640	0.085 ± 0.034	0.277 ± 0.055	0.552 ± 0.324	0.013 ± 0.005	7.13 ± 2.05	0.111 ± 0.084
(n = 10)	Range	0.012–2.089	0.049–0.135	0.175–0.357	0.152–1.382	0.003–0.019	3.56–10.6	0.013–0.226
XT	Mean ±S.D.	0.341 ± 0.299	0.163 ± 0.131	0.187±0.062	0.533 ± 0.222	0.042 ± 0.030	11.6 ± 6.31	0.047 ± 0.021
(n = 8)	Range	0.005–0.691	0.095–0.508	0.106–0.302	0.078–0.856	0.014–0.103	5.52–27.3	0.022–0.094
Total	Mean ± S.D.	0.312 ± 0.434	0.106 ± 0.085	0.336 ± 0.248	0.591 ± 0.297	0.022 ± 0.021	8.83 ± 4.33	0.069 ± 0.060
	Range	0.005–2.089	0.029–0.508	0.106–1.15	0.078–1.382	0.003–0.103	3.56–27.28	0.013–0.226
	MAC ^a^	0.2	1	0.5	10 ^b^	0.2	NV ^c^	0.02

Note: ^a^ MAC, maximum allowable concentrationof heavy metals in rice, recommended by Maximum Levels of Contaminants in Foods (GB 2762 2012); ^b^ Zhao *et al.* (2015); ^c^ NV is the no criterion value.

The brown rice concentration of As in SM ranged from 0.272 to 1.15 mg/kg, with a mean of 0.513 mg/kg, which slightly exceeded the threshold value of 0.5 mg/kg.

### 3.3. Transfer Factor

**Figure 1:**
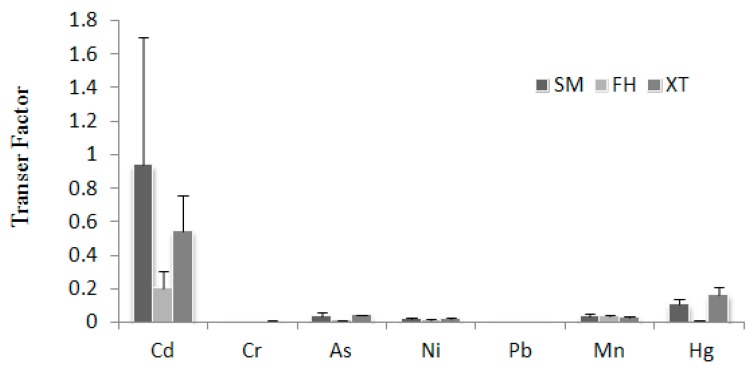
Transfer factor of the seven heavy metals in soil from three study districts.

The transfer factor (TF) for each heavy metal among the three districts is shown in Figure 1. The average values of TF for Cd, Cr, As, Ni, Pb, Mn and Hg decreased in the order of Cd > Hg > Mn > As > Ni > Cr > Pb. Cd had the highest TF of 0.551, followed by Hg with a TF of 0.0853. 

### 3.4. Health Risks to Residents from Heavy Metal Exposure in Brown Rice

The ADI values of heavy metals from brown rice consumption were estimated to be Cd 2.29, Cr 0.78, As 2.45, Ni 4.32, Pb 0.16, Mn 64.7 and Hg 0.50 µg/(kg·d) based upon the assumption that the local population consumes mostly local brown rice. 

The HQs of heavy metals through brown rice consumption for the local residents in Hunan province were Cd 2.29, Cr 0.258, As 8.18, Ni 0.216, Pb 0.045, Mn 0.462 and Hg 3.15 respectively. This suggests that Cd, Hg, and As pose potential non-carcinogenic risks for local people as the HQ values all exceeded one. The other elements had no obvious individual risks. However, the combined HI value of all seven elements was 14.6, implying a high non-carcinogenic health risk for combined exposure to these heavy metals in brown rice. 

CR values of Cd, Cr, As, and Ni were 0.0343, 0.000388, 0.00368, and 0.00393, respectively, all greater than 10^−4^, and CRt was 0.0423, indicating a high potential carcinogenic risk from brown rice consumption.

## 4. Discussion 

Hunan province has a long history of metal mining and smelting. This has promoted economic growth, but, at the same time, released large amounts of heavy metals such as Pb, Zn, and Cd into the environment. Soils from Hunan province are severely polluted by Cd and Hg, with average levels of 1.40 mg/kg and 14.9 mg/kg, which are much higher than the Chinese environmental quality standard for soil, grade II (GB 15618-1995) as well as the corresponding background values of Hunan [36], but lower than the safe limits set by Canada, India and European Union [37]. These findings are consistent with previous studies [15,38,39]. Cd and Hg concentrations showed great variation among the study areas. The variation was associated with the area geology and influenced by human mining activities. For instance, much higher mean levels of Cd and Hg in FH compared with the other two areas could be related to uncontrolled Pb-Zn mining in FH. Most Pb-Zn ores in Hunan province contain associated minerals. Explosive mining processes allowed release of Cd, Hg and other heavy metals and they spread to nearby agricultural soils. This possibility was supported by the fact that heavy metal concentrations in the surface soil were higher than those of sub-surface soils in mining areas [13]. The metal concentrations rapidly decreased with increasing distance from the pollution source [2]. 

Average concentrations of As, Ni, Pb, and Mn were higher than the corresponding background values but were lower than the national standard levels. This result indicated slight pollution of the four elements in local soils and was probably due to agriculture activities. Those activities have been identified as contributors moderately increasing toxic heavy metal pollution through the application of various types of chemical pesticides and fertilizers [40,41]. The As level ranged from 3.16 to 45.2 with a large standard deviation (14.9). This is consistent with the fact that point pollution sources, such as arsenic disulfide mining and slag stacking sites, exist in this county. 

Cd and Hg concentrations in brown rice from three counties, either individual or means, all exceeded the national standard for food safety. The mean Cd level in brown rice was 0.312 mg/kg, which exceeded values from areas influenced by industrial activities such as e-waste processing [42]. The estimated daily intake of Cd through brown rice consumption had been reported to be 35.4 μg/day in Iran [43], 5.38 μg/day in South Korea [44] and 11.7 μg/day in Japan [45]. It was estimated be 600 μg/day in the late 1960s for the local population in the Jinzu river basin in Japan [46]. The calculated daily intake of Cd in this study appeared to be within this range. Rice with Cd levels exceeding 1 mg/kg was termed “Cd rice” [15] and the proportion reached 3.57% in our study. Cd rice would pose a potential health risk to local people [17]. Watanabe reported the association between the Cd concentration in rice and renal dysfunction in individuals living in the Jinzu River basin [47]. The renal dysfunction was associated with an increased mortality in Cd polluted regions [12]. Some patients even developed Itai-itai disease following the excess ingestion of Cd in rice [48]. 

We found high Hg concentrations in brown rice, as did Fang [49] and Meng [50]. The spatial distribution of Hg content in rice samples differed from Hg in the soils. High-Hg rice was distributed in the high-Hg soil areas and in some low-Hg soil areas. For example, the mean soil concentration of Hg in SM and XT was within the safety level, but the mean levels of Hg in brown rice were 0.043 mg/kg and 0.047 mg/kg and in excess of the 0.02 mg/kg national standard. This was also true for As in the soil-rice system of SM. We suggest that the accumulation of heavy metals in rice is not totally a function of soil concentration but is also affected by other factors such as physical-chemical properties of the growing soil. For example, the equilibrium pH value could affect heavy metal sorption and desorption on soil components thus related to transfer ability of metals [51]. Most soils of Hunan are acidic [15], which may promote the transfer of heavy metals from soil to rice. 

The soil-to-rice transfer factor (also called bioaccumulation factor or enrichment index) is an index for evaluating the transfer potential of a metal from soil to plant [25]. It was different among the seven elements. The results suggest that Cd and Hg had relatively higher mobility from soil to rice, followed by Mn, As, Ni, Cr, and Pb, consistent with published reports [3,4]. Soil-to-Plant transfer is a key process for human exposure to toxic heavy metals through the food chain [52]. Because of their mobility, the contamination of Cd and Hg in soil would pose a major heath concern. 

Risk factors, such as HQ and CR are used to assess the potential health risks for humans. HI for brown rice consumption of the seven elements was high and up to 14.6. This indicates a high non-carcinogenic risk from the ingestion of local brown rice. The results are similar to those from a Pb-Zn mining area in Jiangsu province [2] but lower than results from a Pb-acid battery plant in Hunan province [19]. The estimated HI was mainly due to Cd, As, and Hg. Their HQs accounted for 15.7 %, 56.1 %, and 21.6% of the HI. These results indicated that Cd, As, and Hg posed high non-carcinogenic risks to human health. 

Among the seven elements, Cd, Cr, As, and Ni were of greatest concern for their carcinogenicity [21]. According to the USEPA, a one to one hundred in a million chance of additional human cancer over a 70-year lifetime (1.0 × 10^−6^–1.0 × 10^−4^) is regarded as an acceptable or inconsequential risk [30]. The CR_t_ was up to 4.23 × 10^−2^, more than 400× higher than the limit. This demonstrates that consumption of local brown rice poses a potentially great carcinogenic risk. The cancer risk was largely attributed to Cd, which accounted for approximately 81.2%. Long-term environmental exposure to Cd was reported to be associated with an increased risk of all-cancer mortality [16]. A study in the European countries suggested correlations between Cd level and the age-adjusted prostate or breast cancer rates [17]. 

In consideration of the transfer ability of Cd and high concentrations in the soil-rice system, we stress the importance of using technology to reduce Cd levels in rice to ensure food safety in Hunan province.

Although the daily intake of heavy metals through rice is an important exposure pathway, other studies report that humans are also significantly exposed to heavy metals through ingestion of vegetables [53], fish [54] and water [55]. Therefore, the potential health risk of heavy metal exposure is likely to be greater than that estimated in this study. Attention should be focused on the other exposure pathways, such as inhalation and dermal exposure to determine a more precise risk assessment. 

## 5. Conclusions

The mean concentrations of heavy metals in soil were Cd 1.40, Cr 27.2, As 16.8, Ni 33.9, Pb 51.4, Mn 348 and Hg 14.9 mg/kg. The levels of Cd and Hg are much higher than the Chinese environmental quality standard for soil, grade II (GB 15618-1995), which were mainly influenced by uncontrolled mining activities. The slight pollution of As, Ni, Pb, and Mn were probably due to application of chemical pesticides and fertilizers in the process of agricultural production. The mean concentrations of Cd and Hg in brown rice both exceeded the national standard for food safety. The daily intake of Cd was estimated to be 0.133 mg/day in Hunan province and greater than that of Iran and South Korea. The calculated non-carcinogenic risks factor was 14.6 and the carcinogenic risks was 4.23 × 10^−2^, both greater than the limit. In addition, the cancer risk was largely attributed to Cd, which accounted for approximately 81.2%. Therefore, the government should pay more attention to the pollution of Cd in the soil-rice system and apply technology to reduce Cd levels in rice to ensure food safety in Hunan province.

## References

[1] Mohanty M., Pattnaik M.M., Mishra A.K., Patra H.K. (2011). Chromium bioaccumulation in rice grown in contaminated soil and irrigated mine wastewater—A case study at South Kaliapani chromite mine area, Orissa, India. Int. J. Phytoremediation.

[2] Qu C.S., Ma Z.W., Yang J., Liu Y., Bi J., Huang L. (2012). Human exposure pathways of heavy metals in a lead-zinc mining area, Jiangsu Province, China. PLoS ONE.

[3] Zhao K., Fu W., Ye Z., Zhang C. (2015). Contamination and spatial variation of heavy metals in the soil-rice system in Nanxun County, Southeastern China. Int. J. Environ. Res. Public Health.

[4] Marwa E.M., Meharg A.A., Rice C.M. (2012). Risk assessment of potentially toxic elements in agricultural soils and maize tissues from selected districts in Tanzania. Sci. Total Environ..

[5] Pasquini M.W. (2006). The use of town refuse ash in urban agriculture around Jos, Nigeria: Health and environmental risks. Sci. Total Environ..

[6] Hopkins W.G. (1999). Introduction to Plant. Physiology.

[7] Loutfy N., Fuerhacker M., Tundo P., Raccanelli S., El Dien A.G., Ahmed M.T. (2006). Dietary intake of dioxins and dioxin-like PCBs, due to the consumption of dairy products, fish/seafood and meat from Ismailia city, Egypt. Sci. Total Environ..

[8] Phuong T.D., Chuong P.V., Khiem D.T., Kokot S. (1999). Elemental content of Vietnamese rice. Part 1. Sampling, analysis and comparison with previous studies. Analyst.

[9] Cheng F., Zhao N., Xu H., Li Y., Zhang W., Zhu Z., Chen M. (2006). Cadmium and lead contamination in japonica rice grains and its variation among the different locations in southeast China. Sci. Total Environ..

[10] Tsukahara T., Ezaki T., Moriguchi J., Furuki K., Shimbo S., Matsuda-Inoguchi N., Ikeda M. (2003). Rice as the most influential source of cadmium intake among general Japanese population. Sci. Total Environ..

[11] Zhang W.L., Du Y., Zhai M.M., Shang Q. (2014). Cadmium exposure and its health effects: A 19-year follow-up study of a polluted area in China. Sci. Total Environ..

[12] Nogawa K., Kobayashi E., Okubo Y., Suwazono Y. (2004). Environmental cadmium exposure, adverse effects and preventive measures in Japan. Biometals.

[13] Li Y.H., Yang L.S., Li H.R., Wang W.Y., Tang D.Y. (2007). Chemical speciation and pollution characteristics of soil mercury in mercury deposit area of Western Hunan-Eastern Guizhou province. J. Environ. Sci..

[14] Wei C., Wang C., Yang L. (2009). Characterizing spatial distribution and sources of heavy metals in the soils from mining-smelting activities in Shuikoushan, Hunan Province, China. J. Environ. Sci..

[15] Du Y., Hu X.F., Wu X.H., Shu Y., Jiang Y., Yan X.J. (2013). Affects of mining activities on Cd pollution to the paddy soils and rice grain in Hunan province, Central South China. Environ. Monit. Assess..

[16] Wang M., Xu Y., Pan S., Zhang J., Zhong A., Song H., Ling W. (2011). Long-term heavy metal pollution and mortality in a Chinese population: An ecologic study. Biol. Trace. Elem. Res..

[17] Pan J., Plant J.A., Voulvoulis N., Oates C.J., Ihlenfeld C., Pan J. (2010). Cadmium levels in Europe: Implications for human health. Environ. Geochem. Health.

[18] Song D., Zhuang D., Jiang D., Fu J., Wang Q. (2015). Integrated health risk assessment of heavy metals in Suxian county, south China. Int. J. Environ. Res. Public Health.

[19] Cao S., Duan X., Zhao X., Wang B., Ma J., Fan D., Sun C., He B., Wei F., Jiang G. (2015). Health risk assessment of various metal(loid)s via multiple exposure pathways on children living near a typical lead-acid battery plant, China. Environ. Pollut..

[20] Zeng G., Liang J., Guo S., Shi L., Xiang L., Li X., Du C. (2009). Spatial analysis of human health risk associated with ingesting manganese in Huangxing Town, Middle China. Chemosphere.

[21] IARC (2011). Agents classified by the IARC monographs. Oxford Handbook of Occupational Health.

[22] Fitsanakis V.A., Aschner M. (2005). The importance of glutamate, glycine, and gamma-aminobutyric acid transport and regulation in manganese, mercury and lead neurotoxicity. Toxicol. Appl. Pharmacol..

[23] Cloez I., Dumont O., Piciotti M., Bourre J.M. (1987). Alterations of lipid synthesis in the normal and dysmyelinating trembler mouse sciatic nerve by heavy metals (Hg, Pb, Mn, Cu, Ni). Toxicology.

[24] Satpathy D., Reddy M.V., Dhal S.P. (2014). Risk Assessment of heavy metals contamination in paddy soil, plants, and grains (*Oryza sativa* L.) at the East Coast of India. Biomed. Res. Int..

[25] Zheng N., Wang Q.C., Zheng D.M. (2007). Health risk of Hg, Pb, Cd, Zn, and Cu to the inhabitants around Huludao Zinc Plant in China via consumption of vegetables. Sci. Total Environ..

[26] Xiao J. (2007). Analysis of Dietary Structure and Nutrient Intake Status of Adult of Hunan Province.

[27] Gu D., He J., Duan X., Reynolds K., Wu X., Chen J., Huang G., Chen C.S., Whelton P.K. (2006). Body weight and mortality among men and women in China. JAMA.

[28] FAO/WHO Summary and Conclusions of the 61st Meeting of the Joint FAO/WHO Expert Committee on Food Additives. ftp://ftp.fao.org/es/esn/jecfa/jecfa61sc.pdf.

[29] EPA Integrated Risk Information System. http://www.epa.gov/iris/rfd.htm.

[30] EPA (2011). Risk Assessment Guidance for Superfund (Part E, Part F).

[31] Al-Saleh I., Nester M., DeVol E., Shinwari N., Al-Shahria S. (1999). Determinants of blood lead levels in Saudi Arabian schoolgirls. Int. J. Occup. Environ. Health.

[32] EPA (1986). Guidelines for the health risk assessment of chemical mixtures. Fed. Regist..

[33] EPA (1989). Risk Assessment Guidance for Superfund: Volume I. Human Health Evaluation Manual (Part A). U.S..

[34] NEPAC (1995). Environmental Quality of Standard for Soils.

[35] NEPAC (2012). Maximum Levels of Contaminants in Foods.

[36] Pan Y., Yang G. (1988). Soil Background Value in Hunan and Study Methods.

[37] Orisakwe O.E., Nduka J.K., Amadi C.N., Dike D.O., Bede O. (2012). Heavy metals health risk assessment for population via consumption of food crops and fruits in Owerri, South Eastern, Nigeria. Chem. Cent. J..

[38] Zhang X., Zhong T., Liu L., Ouyang X. (2015). Impact of soil heavy metal pollution on food safety in China. PLoS ONE.

[39] Zhou H., Zeng M., Zhou X., Liao B.H., Liu J., Lei M., Zhong Q.Y., Zeng H. (2013). Assessment of heavy metal contamination and bioaccumulation in soybean plants from mining and smelting areas of southern Hunan Province, China. Environ. Toxicol. Chem..

[40] Saha N., Rahman M.S., Jolly Y.N., Rahman A., Sattar M.A., Hai M. A. (2015). Spatial distribution and contamination assessment of six heavy metals in soils and their transfer into mature tobacco plants in Kushtia District, Bangladesh. Environmental Science and Pollution Research.

[41] Liu M.X., Yang Y.Y., Yun X.Y., Zhang M.M., Wang J. (2015). Concentrations, distribution, sources, and ecological risk assessment of heavy metals in agricultural top soil of the Three Gorges Dam region China. Environ. Monit. Assess..

[42] Fu J., Zhang A., Wang T., Qu G., Shao J., Yuan B., Wang Y., Jiang G. (2013). Influence of e-waste dismantling and its regulations: Temporal trend, spatial distribution of heavy metals in rice grains, and its potential health risk. Environ. Sci. Technol..

[43] Chamannejadian A., Sayyad G., Moezzi A., Jahangiri A. (2013). Evaluation of estimated daily intake (EDI) of cadmium and lead for rice (*Oryza sativa* L.) in calcareous soils. Iranian J. Environ. Health Sci. Eng..

[44] Jung M.C., Yun S.T., Lee J.S., Lee J.U. (2005). Baseline study on essential and trace elements in polished rice from South Korea. Environ. Geochem. Health.

[45] Watanabe T., Zhang Z.W., Moon C.S., Shimbo S., Nakatsuka H., Matsuda-Inoguchi N., Higashikawa K., Ikeda M. (2000). Cadmium exposure of women in general populations in Japan during 1991–1997 compared with 1977–1981. Int. Arch. Occup. Environ. Health.

[46] Ikeda M., Ezaki K., Moriguchi T.T.J. (2004). Dietary cadmium intake in polluted and non-polluted areas in Japan in the past and in the present. Int. Arch. Occup. Environ. Health.

[47] Watanabe Y., Kobayashi E., Okubo Y., Suwazono Y., Kido T., Nogawa K. (2002). Relationship between cadmium concentration in rice and renal dysfunction in individual subjects of the Jinzu River basin determined using a logistic regression analysis. Toxicology.

[48] Kobayashi E., Suwazono Y., Dochi M., Honda R., Kido T. (2009). Influence of consumption of cadmium-polluted rice or Jinzu River water on occurrence of renal tubular dysfunction and/or Itai-itai Disease. Biol. Trace. Elem. Res..

[49] Fang Y., Sun X., Yang W., Ma N., Xin Z., Fu J., Liu X., Liu M., Mariga A.M., Zhu X. (2014). Concentrations and health risks of lead, cadmium, arsenic, and mercury in rice and edible mushrooms in China. Food Chem..

[50] Meng B., Feng X., Qiu G., Anderson C.W., Wang J., Zhao L. (2014). Localization and speciation of mercury in brown rice with implications for pan-Asian public health. Environ. Sci. Technol..

[51] Najafi S., Jalali M. (2015). Effects of organic acids on cadmium and copper sorption and desorption by two calcareous soils. Environ. Monit. Assess..

[52] Zhuang P., McBride M.B., Xia H., Li N., Li Z. (2009). Health risk from heavy metals via consumption of food crops in the vicinity of Dabaoshan mine, South China. Sci. Total. Environ..

[53] Rahman M.A., Rahman M.M., Reichman S.M., Lim R.P., Naidu R. (2014). Heavy metals in Australian grown and imported rice and vegetables on sale in Australia: Health hazard. Ecotoxicol. Environ. Saf..

[54] Onsanit S., Ke C., Wang X., Wang K.J., Wang W.X. (2010). Trace elements in two marine fish cultured in fish cages in Fujian province, China. Environ. Pollut..

[55] Ngueta G., Prevost M., Deshommes E., Abdous B., Gauvin D., Levallois P. (2014). Exposure of young children to household water lead in the Montreal area (Canada): The potential influence of winter-to-summer changes in water lead levels on children’s blood lead concentration. Environ. Int..

